# Synergistic and Antagonistic Activity of Selected Dietary Phytochemicals against Oxidative Stress-Induced Injury in Cardiac H9c2 Cells via the Nrf2 Signaling Pathway

**DOI:** 10.3390/foods13152440

**Published:** 2024-08-02

**Authors:** Jingwen Yu, Xiangwei Xiao, Baiying Chen, Zeyuan Deng, Xuan Chen, Yawei Fan, Hongyan Li

**Affiliations:** 1 State Key Laboratory of Food Science and Resources, Nanchang University, Nanchang 330047, China; 13123615891@163.com (J.Y.); xxw18779665639@163.com (X.X.); chenbaiyinghappy@foxmail.com (B.C.); zeyuandeng@hotmail.com (Z.D.); xuanchen2206@hotmail.com (X.C.); yaweifan@ncu.edu.cn (Y.F.); 2 Institute for Advanced Study, Nanchang University, Nanchang 330031, China

**Keywords:** phytochemical mixtures, antioxidant interactions, synergistic, antagonistic

## Abstract

The antioxidant activities of lycopene (LY), lutein (LU), chlorogenic acid (CA), and delphinidin (DP) were tested in vitro on H9c2 cell-based models. Some indicators, such as the generation of reactive oxygen (ROS), the quantification of cell antioxidant activity (CAA), and the expressions of SOD, GSH-Px, and CAT, were calculated to examine their antioxidant interactions. From our results, the phytochemical mixtures (M1: CA-LU: F3/10, M2: DP-CA: F7/10, M3: DP-LY: F5/10) displayed strong synergistic effects based on the generation of ROS and the quantification of CAA. However, great antagonistic bioactivities were seen in the combinations of LY-LU: F5/10 (M4), CA-LU: F9/10 (M5), and DP-LY: F7/10 (M6). Western blotting analysis indicated that the possible mechanism underlying the synergistic antioxidant interactions among phytochemical combinations was to enhance the accumulation of Nrf2 in the nucleus and the expression of its downstream antioxidant enzymes, HO-1 and GCLC. The combinations (M1–M3 groups) showed significant protection against the loss of mitochondrial membrane potential than individual groups to avoid excessive ROS production. The M4–M6 groups exerted antagonistic protective effects compared with the individual groups. In addition, lutein and lycopene absorption was improved more because of the presence of chlorogenic acid and delphinidin in the M1 and M3 groups, respectively. However, delphinidin significantly reduced the cellular uptake of lycopene in the M6 group. It appeared that antioxidant interactions of phytochemical combinations may contribute to the restoration of cellular redox homeostasis and lead to an improvement in diet quality and collocation.

## 1. Introduction

With the increase in population aging, the prevalence of cardiovascular diseases (CVDs) keeps increasing, and accumulating evidence highlights the pivotal role of mitochondria and ROS in CVDs [1,2]. The great interest in studying phytochemicals is due to their powerful ability to deactivate reactive oxygen species (free radicals, singlet oxygen, and peroxides) and chelate metal ions [3]. Indeed, in a multiethnic cohort study, phytochemicals showed good potential effects in preventing arteriosclerosis, protecting the heart and brain, anti-diabetic and lipoprotein metabolism, and relaxing blood vessels [4,5].

Lycopene and lutein are major lipophilic carotenoids, while chlorogenic acid and delphinidin are hydrophilic phenolic antioxidants. They are widespread in vegetables and fruits; thus, they are considered the main contributors to preventing chronic diseases. In addition, they are often co-presented in a typical human diet that is composed of plant-based foods. Therefore, they can interact with each other during digestion and absorption to result in antagonism (lower effects than addition), addition, or synergy (greater effects than addition) on antioxidant activities. Previous studies showed that the overuse of a single high dosage of antioxidant supplements and β-carotene may increase toxicity and mortality [6]. In comparison, antioxidants present in fruits and vegetables have lower toxicity to the body, and the synergistic effect that exists among them may further enhance their antioxidant activities. Therefore, it is meaningful to study the antioxidant activities of phytochemical combinations. 

In recent years, some studies focused on the antioxidant interactions among phytochemicals have emerged. For instance, combinations of lycopene and methoxylated anthocyanins induced a synergistic effect, where the anti-inflammatory impact on cytokine IL-8 inhibition was enhanced by 15–69% [7]. By the cellular antioxidant activity (CAA) assay, the combinations of Punica granatum and Malus domestica at the ratio of 3:1 and 1:1 both showed synergistic effects [8]. Our previous studies [9,10] reported that when the total concentration was constant, antioxidant interactions between hydrophilic and lipophilic phytochemicals were associated with their ratios. 

These studies showed that when phytochemicals were mixed in different proportions, their antioxidant activities were different from that of a single substance. In recent years, exploring antioxidant substances with synergistic effects and revealing their potential interaction mechanisms have become important issues in food areas. Nevertheless, there is little research to date on antioxidant interactions with hydrophilic and lipophilic antioxidants. Therefore, in this study, lipophilic carotenoids (lycopene and lutein) and hydrophilic phenolics (chlorogenic acid and delphinidin) were selected to study the antioxidant interactions by the ROS level, the activity of the antioxidant enzyme (SOD, CAT, and GSH-Px), the expression of Nrf2, HO-1, and GCLC, the differences in mitochondrial membrane potential, and the degradation and absorption of carotenoids in H9c2 cells. 

## 2. Materials and Methods

### 2.1. Materials

Delphinidin was purchased from Phytolab Co., Ltd. (Shanghai, China). Lycopene, lutein, and chlorogenic acid were purchased from Aladdin Reagent Co., Ltd. (Shanghai, China). The purity of these phytochemicals was up to 98%. Dimethyl sulfoxide and tetrahydrofuran were from Damao Co., Ltd. (Tianjing, China). The rat cardiomyocytes line H9c2 was obtained from Procell Life Science & Technology Co., Ltd. (Wuhan, China). Dulbecco’s modified eagle’s medium (DMEM) and fatal bovine serum (FBS) were obtained from Biological Industries, Shanghai, China. H_2_O_2_ was purchased from Sinopharm Group Chemical Reagent Co., Ltd. (Beijing, China). 3-(4, 5-Dimethylthiazole-2-yl)-2, 5 diphenyltetrazolium bromide (MTT) was procured from Sigma Chemical Co. (St. Louis, MO, USA). 

### 2.2. Methods

#### 2.2.1. Phytochemical Stock Preparation

Stock solutions of lycopene and lutein (5 mmol/L) were prepared in tetrahydrofuran and stored at −20 °C. Stock solutions of chlorogenic acid and delphinidin (5 mmol/L) were prepared in dimethyl sulfoxide and stored at −4 °C for no longer than 1 week. For further research, these stock solutions were diluted in different ratios in a cell culture medium.

#### 2.2.2. General Cell Culture Condition

The rat cardiomyocytes line H9c2 was well maintained in a complete growth medium containing the following: Dulbecco’s modified Eagle’s medium (DMEM, 01-0511 ACS, Biological Industries, Shanghai, China), 10% fetal bovine serum (FBS, Biological Industries, Shanghai, China), 1% nonessential amino acid (Solarbio Co., Beijing, China), and anti-microbial agents (penicillin and streptomycin: 1%, Solarbio Co., Beijing, China). The cells were incubated at 37 °C and 5% CO_2_, and the media were changed daily. The cells were grown in 25 cm^2^ plastic flasks (BIOFIL^®^, Jet Bio-Filtration Co., Guangzhou, China) in a CO_2_ incubator (HERACELL 150i, Thermo Fisher Scientific Inc., Shanghai, China) at 37 °C and 5% CO_2_. When the cells achieved 80–90% confluence, they were subcultured.

#### 2.2.3. Cell Viability

The cell viability assay was determined using the MTT method. Briefly, H9c2 cells were seeded on a 96-well plate for 200 μL (1 × 10^4^ cells per well) and treated with phytochemicals diluted to different concentrations with culture media for 12 h at 37 °C, 5% CO_2_. After the treatment, the medium was discarded, and the cells were washed twice with phosphate-buffered saline (PBS). A total of 200 μL of 0.5 mg/mL MTT was added into each well, and the samples continued to develop after 4 h at 37 °C. Then, 150 μL of DMSO was added to dissolve the purple formazan crystals after the supernatant was removed. After the plates were gently shaken for 10 min at room temperature in the dark, the formed formazan crystals were quantified, and the absorbance was measured at a wavelength of 490 nm in a microplate reader (Thermo Scientific Varioskan Flash, Vantaa, Finland). Only preparations with cell viability greater than 95% (of control) were employed for subsequent experiments. At least three tests were performed on each sample.

#### 2.2.4. Cellular Antioxidant Activity (CAA) Assay

The CAA assay developed by Wolfe and Liu [11] was used with slight modification. The H9c2 cells were seeded on a 96-well plate with 100 μL of cell suspension (6 × 10^4^ cells per well). After incubation for 24 h, the growing medium was discarded and treated for 1 h with 100 μL of phytochemicals at 37 °C and 5% CO_2_. Then, 25 μM 2′,7′-dichlorodihydrofluorescein diacetate (DCFH-DA) prepared in DMEM with H_2_O_2_ (150 μmol/L) was added. The 96-well plate was placed into a Fluoroskan Ascent FL platereader (Thermo Lab systems, Franklin, MA, USA) at 37 °C and was read at λ excitation = 485 nm and λ emission = 520 nm every 5 min for 1 h. The cellular antioxidant activity (CAA) was calculated as:

CAA unit = 1 − AUC sample − AUC control/AUC H_2_O_2_ − AUC control

where AUC sample is the integrated area under the sample fluorescence, AUC H_2_O_2_ is the integrated area from the H_2_O_2_ curve, and AUC control is the integrated area from the control curve versus the time curve.

In this study, five different fractions (1/10, 3/10, 5/10, 7/10, and 9/10) of each phytochemical (lycopene, lutein, chlorogenic acid, and delphinidin) were combined with various fractions of another one (9/10, 7/10, 5/10, 3/10, and 1/10). The interaction types among the binary mixes of phytochemicals were simulated using the combination index (CI) computed by the CompuSyn program. For binary combinations at a% activity:

CIa%=CA/ICa(A)+CB/ICa(B)

where CIa is the combination index for the binary mixture at a% activity, ICa (A) and ICa (B) represent the single doses of compound A and compound B that provide a% activity, and CA and CB represent the proportionate doses of compound A and compound B, respectively. CI < 1 indicates synergy; CI = 1 indicates addition; and CI > 1 indicates antagonism. 

#### 2.2.5. Reactive Oxygen Species (ROS) Production

The formation of ROS in the cells was measured by the ROS assay kit (Beyotime Biotechnology, Shanghai, China) according to the instructions of the manufacturer. H9c2 cells were seeded on 6-well plates at 5 × 10^5^ cells/well for 12 h. The cells were cultured and treated with the phytochemicals. After 12 h of incubation with the phytochemicals at 37 °C and 5% CO_2_, 2 mL of H_2_O_2_ (final concentration 150 μM) was added into each well, and the plate was incubated for 30 min. The cells were washed with phosphate-buffered saline (PBS) followed by an addition of 500 μL of 5 μM DCFH-DA and then further incubated in the dark for 20 min. Next, the medium was discarded, and the cells were washed three times with a serum-free medium. The cell pellets were centrifuged at 1000 rpm for 5 min and re-suspended in PBS. The intracellular fluorescence was measured by flow cytometry (BD FACS, Becton Dickinson Co., Shanghai, China) withCellQuest (version 3.1). The percentages of cells in different phases of the cell cycle within the GFP-positive population were analyzed using the FlowJo software (version 7.6.1).

#### 2.2.6. The Activity of SOD, CAT, and GSH-Px Enzymes

The H9c2 cells were seeded at 5 × 10^5^ cells/well on a Petri dish for 12 h. After pretreating with the phytochemicals for 12 h, the cells were added with 150 μM H_2_O_2_ for 1 h incubation to induce oxidative stress. After that, the cells were lysed with RIPA lysis buffer containing protease inhibitors (Beyotime Biotech, Shanghai, China) and PMSF (Sigma Chemical, St. Louis, MO, USA), and the concentration of protein was determined by the BCA protein assay (Beyotime Biotech, Shanghai, China). Then, the cells were collected to quantify the superoxide dismutase (SOD), catalase assay (CAT), and glutathione peroxidase (GSH-Px) activity with the corresponding assay kits according to the instructions of Beyotime Biotechnology (Shanghai, China). The change in absorbance was converted to units of enzyme activity standardized to milligram protein from a standard curve. 

#### 2.2.7. Western Blot Analysis for Nrf2, HO-1, and GCLC Expression

Proteins in H9c2 cells were extracted with RIPA lysis buffer containing protease inhibitors and PMSF, and the protein concentrations were counted by the BCA protein assay. Equal amounts of proteins were loaded and separated by 8% sodium dodecyl-sulfate polyacrylamide gel electrophoresis (SDS-PAGE) and transferred to polyvinylidene fluoride (PVDF) membranes (Roche Diagnostics GmbH, Mannheim, Germany). A total of 5% non-fat milk diluted in Tris-buffered saline–Tween-20 (TBST) was used to block the PVDF membranes for 2 h at room temperature. The membranes were incubated overnight at 4 °C with appropriate primary antibodies and then were combined with the secondary antibodies at room temperature for 2 h. The experiment involved primary antibodies against Anti-GCLC (ab190685, Abcam, Cambridge, UK), Anti-Nrf2 (ab31163), and Anti-Heme Oxygenase 1 (ab68477). Two kinds of second antibodies (goat anti-rabbit (HS201-01, TransGen Biotech, Beijing, China) and goat anti-mouse (HS101-01)) were used. Finally, chemiluminescence (ECL) reagent (Beyotime Biotechnology, Shanghai, China) was used to visualize the bands of the proteins, and quantitative analysis of different proteins was performed by Image Laboratory 2004 software (version 6.1).

#### 2.2.8. Immunofluorescence Staining for the Localization of Nrf2 in Cells

Immunofluorescence (IF) staining was used to examine the expression of Nrf2 in the cells. Briefly, H9c2 cells were collected, washed with PBS buffer, and fixed with 4% paraformaldehyde (Solarbio Co., Beijing, China) at room temperature for 30 min. After fixation, the cells were washed with PBS three times, permeabilized with 0.4% Triton X-100 (Solarbio Co., Beijing, China) for 15 min, and then incubated with 5% BSA (Solarbio Co., Beijing, China) for 30 min at room temperature to block non-specific antibody binding. After that, the cells were incubated with primary Nrf2 antibody diluted in 1% BSA in a humidified chamber overnight at 4 °C. After washing in PBS, the cells were incubated with a mixture of secondary antibodies with FITC fluorochrome at a dilution of 1:500 in 1% BSA (Trans Biotechnology, Beijing, China) for 1 h at room temperature in the dark. The mixture solution was decanted, and nuclear morphology was counterstained with DAPI dye (Beyotime Biotechnology, Shanghai, China) for 10 min and then rinsed with PBS. Images of the labeled cells were taken under a microscope, and double-stained cells were photographed using an inverted fluorescence microscope (Nikon-Ti-U, Shanghai, China).

#### 2.2.9. The Effects of the Inhibitor ATRA on Nrf2, HO-1, and GCLC Expression

In order to explore the effects of phytochemical combinations on Nrf2 pathway activation, all-trans retinoic acid (ATRA) was used to inhibit the Nrf2 pathway and observe the expression of Nrf2, HO-1, and GCLC. The H9c2 cells were seeded at 5 × 10^5^ cells/well on a Petri dish. Twelve hours after seeding, the growth medium was removed, and the dish was washed with PBS. The cells were treated for 12 h with 3 mL of phytochemical compounds plus 1 µM ATRA dissolved in a growth medium. After that, the cells were added with 150 μM H_2_O_2_ for a 1 h incubation to induce oxidative stress. The proteins in the H9c2 cells were extracted and used to analyze the protein expression of Nrf2, HO-1, and GCLC by Western blotting, as described above.

#### 2.2.10. JC-1 Staining for Membrane Potential Differences in the Mitochondrial Function of H9C2 Cells

The mitochondrial probe 5,5′,6,6′-tetrachloro-1,1′,3,3′-tetraethylbenzimidazolyl-carbocyanine iodide (JC-1) not only identifies mitochondria exhibiting low membrane potentials by the emission of green fluorescence (range, 510–520 nm) but JC-1 also forms aggregate at high membrane potentials emitting, a bright red-orange fluorescence at 590 nm [12]. The cells were seeded in a 12-well culture plate for 24 h and treated with phytochemicals for 12 h; after that, the cells were added with 150 μM H_2_O_2_ for a 1 h incubation to induce oxidative stress. Then, the cells were washed with PBS and incubated in 10 µg/mL JC-1 for 20 min at 37 °C. The cells were washed with incubation buffer three times. The inverted fluorescence microscope was used to analyze membrane potentials across mitochondria in the H9C2 cells.

#### 2.2.11. Degradation and Absorption of Lycopene and Lutein in H9c2 Cells

A total of 5 μM lycopene or lutein prepared with DMEM medium was divided into several centrifuge tubes and incubated at 37 °C, 5% CO_2_. These tubes were taken out at 0, 1, 2, 4, 6, 10, and 12 h and then filtered through a 0.45 μm filter for further analysis.

H9c2 cells (5 × 105 cells per well) were incubated with phytochemicals for 12 h. Following the incubation, the growth medium was removed, and the cells were washed with cold PBS 2–3 times. Then, the cells were lysed with RIPA lysis buffer containing protease inhibitors and PMSF on ice for 30 min and centrifuged at 14,000 rpm for 15 min at 4 °C. A total of 15 μL supernatants were used to measure the protein concentration. After adding 200 μL acetone, the mixture was repeatedly shaken 2–3 times. The supernatant was gathered and stored at −80 °C after evaporating to dryness with nitrogen. The extracts were redissolved in dichloromethane and mixed until completely dissolved. After filtration through a 0.45 μm filter, the extracts were ready for analysis. 

The content of lycopene was determined by the Agilent 1100 HPLC system. The analysis conditions were as follows: the liquid chromatography column was Agilent Eclipse XDB C18 column, the mobile phase consisted of 35% methanol, 35% acetonitrile, and 30% dichloromethane, the column temperature was controlled at 30 °C, the DAD detection wavelength was 472 nm, the sample injection volume was 10 µL, the flow rate was 1 mL/min, the running time was 20 min, and the post running time was 2 min. 

For lutein, the mobile phase consisted of (A) methanol and (B) 0.1% formic acid–acetonitrile. The gradient program was as follows: 0–3 min 50–60% B, 3–8 min 60–70% B, 8–10 min 70–80% B, and 10–20 min, 80–90% B. The DAD detection wavelength was 460 nm; the other conditions were set as above.

#### 2.2.12. Statistical Analysis

Every experiment was conducted in at least three duplicates. The study data were reported as means ± standard deviations. Statistical analyses were carried out a one-way analysis of variance (ANOVA) followed by Duncan’s multiple range tests using a statistical software package (SPSS version 18.0). (*p*-values < 0.05 were considered statistically significant. The analysis and graphs were carried out using GraphPad Prism (version 9.5.0).

## 3. Results

### 3.1. Cell Viability

The H9c2 cell cytotoxicity of each tested phytochemical was assessed prior to the biological assays. Compared with the control group, when the concentrations of LY and LU were above 5 μM, the concentration of CA was above 27 μM, and DP was above 10 μM, the cell viability reduced (Figure S1, *p* < 0.05). Therefore, the appropriate concentration for the individual or combined phytochemicals tested at 5 μM (cell relative viability was greater than 95%) was chosen for subsequent experiments.

### 3.2. Cellular Antioxidant Activity (CAA)

Compared with the H_2_O_2_-induced group, the cellular ROS levels treated with these four phytochemicals were reduced. When LY, LU, and CA were in specific concentration ranges (LY < 3 µM, LU < 2.5 µM, CA < 18µM, respectively), ROS generation was remarkably decreased (*p* < 0.05), and intracellular ROS generation increased when their concentrations were further increased (Figures S2 and S3). Particularly, when the concentration of DP increased from 0.5 µM to 10 µM, the ROS level of H9c2 cells decreased significantly (*p* < 0.05), which meant that the cellular antioxidant activity of DP was dose-dependent.

The combination index (CI) of different fractions of phytochemicals at different ratios was calculated to analyze their antioxidant interactive effects, and the results are presented in Table 1. The radical scavenging activity was expressed as CI_25_, which indicated the CI value when phytochemicals reduced the generation of ROS at the scavenging capacity of 25%. Among phytochemical combinations, F3/10 CA-LU, F7/10 DP-CA, and F5/10 DP-LY showed lower CI values (0.27 ± 0.15, 0.53 ± 0.01, and 0.57 ± 0.04, respectively), which indicated stronger synergistic inhibitions on intracellular ROS generation (CI < 1). However, some combinations showed strong antagonistic effects. For instance, F5/10 LY-LU had the maximum CI_25_ value (22.85 ± 1.93), followed by F9/10 CA-LU (6.40 ± 5.01) and F7/10 DP-LY (4.54 ± 1.52). Based on the results of CAA, six combinations [CA-LU: F3/10 (M1), DP-CA: F7/10 (M2), DP-LY: F5/10 (M3), LY-LU: F5/10(M4), CA-LU: F9/10 (M5), DP-LY: F7/10 (M6)] with significant interactional effects were chosen to further investigate the possible mechanisms of the antioxidant interactions (synergy or antagonism).

### 3.3. Interaction Effects of Phytochemical Combinations on the Inhibition of Intracellular ROS Generation

The ROS level in the H_2_O_2_-induced group was five-fold higher than that in the control group (Figure S4, *p* < 0.05). When the cells were incubated with the individual phytochemicals, intracellular ROS production was much lower in the cells treated with individual phytochemicals than in the H_2_O_2_-induced group (*p* < 0.05). What is more, combined phytochemicals (M1, M2, and M3 groups) showed synergistically inhibited effects on intracellular ROS generation. However, the M4 and M5 groups showed antagonistic effects. For example, the ROS level in the M1 group was 223.91 ± 13.28, which was lower than those in CA (323.43 ± 8.58) and LU (353.56 ± 21.64). The ROS generation in the M4 group was 328.24 ± 23.29, while the ROS generation in the LY and LU groups was 282.41 ± 16.58 and 353.56 ± 21.64, respectively. 

### 3.4. Interaction Effects of Phytochemical Combinations on SOD, GSH-Px, and CAT Enzyme Activities

The H_2_O_2_-induced group had a significant decrease in the activities of SOD and CAT compared with the control group (Figure S5, *p* < 0.05). When compared with the control group, the activities of SOD and CAT of the H_2_O_2_-induced group were significantly lower. Compared with the H_2_O_2_-induce group, the activity of GSH-Px was enhanced obviously in the individual CA and LY groups and the activity of CAT was enhanced obviously in the individual DP, CA, and LY groups (*p* < 0.05). Among phytochemical combinations, the SOD activity was significantly increased in M2 and M3 and the CAT activity was significantly increased in M1 (*p* < 0.05). This means that three combination groups (M1, M2, M3) showed synergistic effects on the recovery of SOD and CAT depletion. However, the M4, M5, and M6 groups did not show antagonistic effects on SOD. As for GSH-Px activities, M1 and M4 groups had significant antioxidant synergistic and antagonistic effects, respectively. In addition, the other groups showed no significant differences in the expression of GSH-Px. Interestingly, some phytochemical combinations had different effects on different antioxidant enzyme activities. For instance, antagonism effects on GSH-Px activities were shown in the M3 group. However, it had a significant antioxidant synergistic effect on CAT enzyme activities. This means that the expression of antioxidant enzymes was less sensitive to the antioxidant interactions induced by phytochemical interactions (M1, M2, M3, M4, M5, and M6 groups).

### 3.5. Interaction Effects of Phytochemical Combinations on the Expression of Nrf2 and Its Downstream Antioxidant Proteins

**Figure 1:**
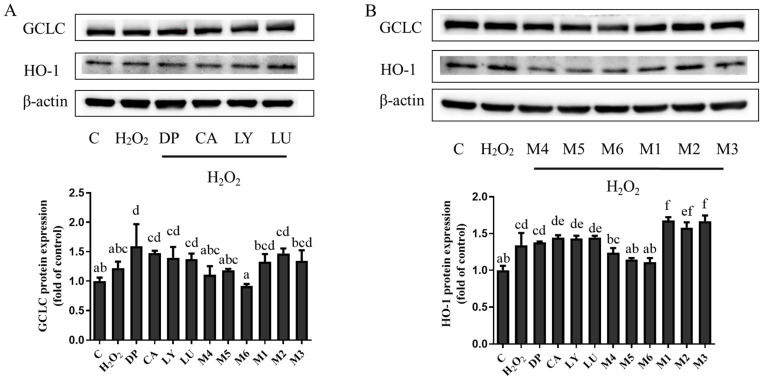
Effects of the pretreatments with individual phytochemicals and combinations on guidance control launch console (GCLC) and Heme oxygenase 1(HO-1) protein expression in H9c2 cells. (**A**) The cells were treated with delphinidin, chlorogenic acid, lycopene, lutein, or (**B**) their combinations. The figure shows the immunoblot and densitometric analysis of one experiment representing GCLC and HO-1 expression in H9c2 cells. β-actin was used as the internal control in the cytosolic protein. DP—delphinidin, CA—chlorogenic acid, LY—lycopene, LU—lutein, M1: CA-LU F3/10, M2: DP-CA F7/10, M3: DP-LY F5/10, M4: LY-LU F5/10, M5: CA-LU F9/10, and M6: DP-LY F7/10. Where CA-LU F1/10 refers to the quantity of chlorogenic acid (1/10) to lutein (9/10) in the binary mixtures. Values with different letters (a–f) indicate that the GCLC and HO-1protein expressions in each group were significantly different (*p* < 0.05).

The effects of phytochemical-induced Nrf2 signaling were investigated by the expression of two Nrf2-target downstream proteins, namely, GCLC and HO-1 (Figure 1). The results of the Western blot assay showed that the protein expression of GCLC and HO-1 was slightly enhanced in the individual phytochemical groups. It was observed that the protein expression of GCLC was attenuated in the M4, M5, and M6 groups treated with phytochemical combinations. Among the combinations, the M6 group (DP: LY = 7:3) showed significantly less GCLC protein expression than individual DP and LY (*p* < 0.05). The protein expression of HO-1 in the M1, M2, and M3 groups increased dramatically, while it decreased markedly in the M4, M5, and M6 groups.

**Figure 2:**
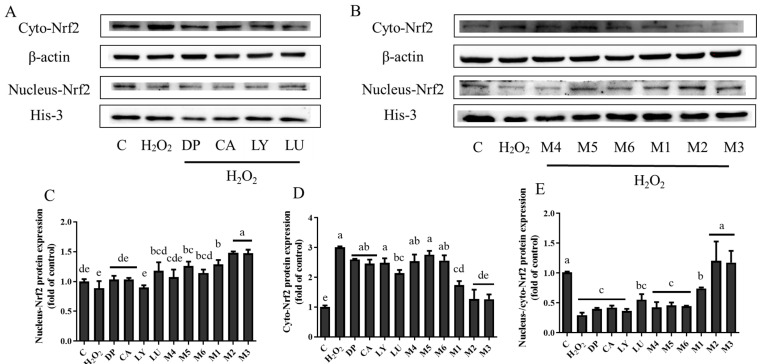
The effects of the pretreatments with individual phytochemicals and combinations of phytochemicals on cyto-Nrf2 and nucleus-Nrf2 protein expression in H9c2 cells. (**A**) The cells were treated with delphinidin, chlorogenic acid, lycopene, lutein, or (**B**) their combinations. The protein expression of cyto-Nrf2 (**C**), nucleus-Nrf2 (**D**), and their ratios (**E**) were analyzed by Western blot. β-actin and His-3 were used as the internal control in the cytosolic and nuclear proteins, respectively. DP—delphinidin, CA—chlorogenic acid, LY—lycopene, LU—lutein, M1: CA-LU F3/10, M2: DP-CA F7/10, M3: DP-LY F5/10, M4: LY-LU F5/10, M5: CA-LU F9/10, M6: DP-LY F7/10. Where CA-LU F1/10 refers to the quantity of chlorogenic acid (1/10) to lutein (9/10) in the binary mixtures. Values with different letters (a–e) indicate that the cyto-Nrf2 or/and nucleus-Nrf2 protein expression in each group was significantly different (*p* < 0.05).

**Figure 3:**
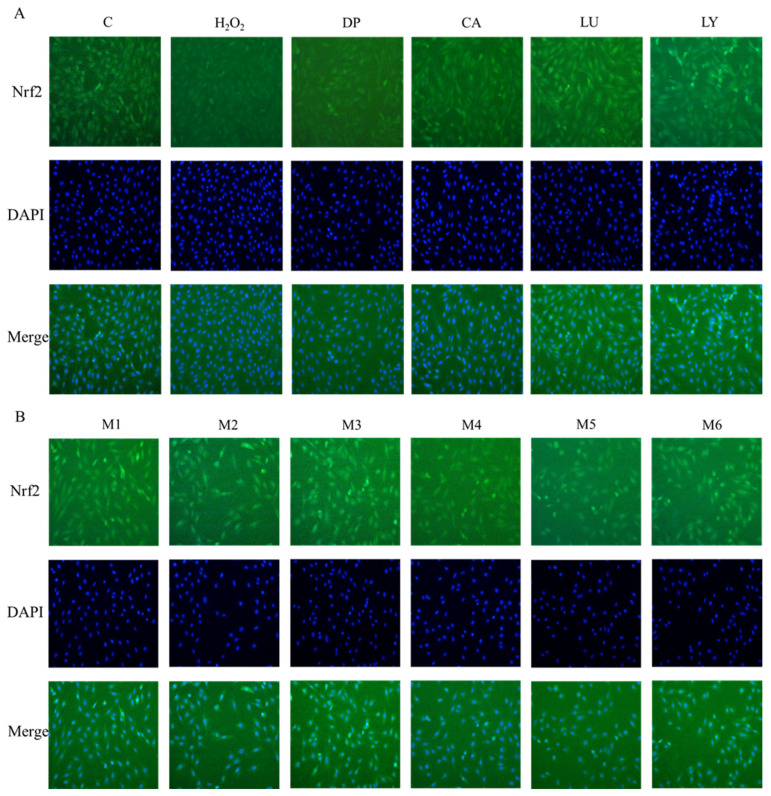
Immunofluorescence staining in H9c2 cells pretreated with individual and phytochemical combinations. (**A**) The cells were treated with delphinidin, chlorogenic acid, lycopene, lutein, or (**B**) their combinations. The immunofluorescence assay evidenced that H_2_O_2_ promoted the nuclear translocation of nuclear factor erythroid 2-related factor 2 (Nrf2) in H9c2 cells. Nrf2 protein was visualized with Nrf2 antibody and FITC-labeled antibody, and the nuclear morphology was visualized with DAPI dye. DP—delphinidin, CA—chlorogenic acid, LU—lutein, LY—lycopene, M1: CA-LU F3/10, M2: DP-CA F7/10, M3: DP-LY F5/10, M4: LY-LU F5/10, M5: CA-LU F9/10, M6: DP-LY F7/10. Where CA-LU F1/10 refers to the quantity of chlorogenic acid (1/10) to lutein (9/10) in the binary mixtures.

An increasing amount of data suggests that Nrf2 accumulation in the nucleus of cell lysates might occur concurrently with Nrf2 activation [13]. The Western blot analysis revealed that the M1, M2, and M3 groups had an accumulation of Nrf2 in the nucleus fraction and a decrease in Nrf2 in the cytoplasmic fraction (Figure 2). In addition, the immunostaining of Nrf2 expression was performed to confirm the location of Nrf2 (Figure 3). The translocation of Nrf2 from the cytosol to the nucleus was apparently higher in cells that were pretreated with phytochemicals of the M1, M2, and M3 groups. However, there was no difference in the level of Nrf2 protein in the nucleus in any of the other groups. These results demonstrated that the levels of Nrf2 in the nucleus and the expression of its downstream antioxidant proteins were significantly higher in the phytochemical combinations treatment of the M1, M2, and M3 groups than those in the other groups. 

### 3.6. The Effects of the Inhibitor ATRA on Nrf2, HO-1, and GCLC Protein Expression

**Figure 4:**
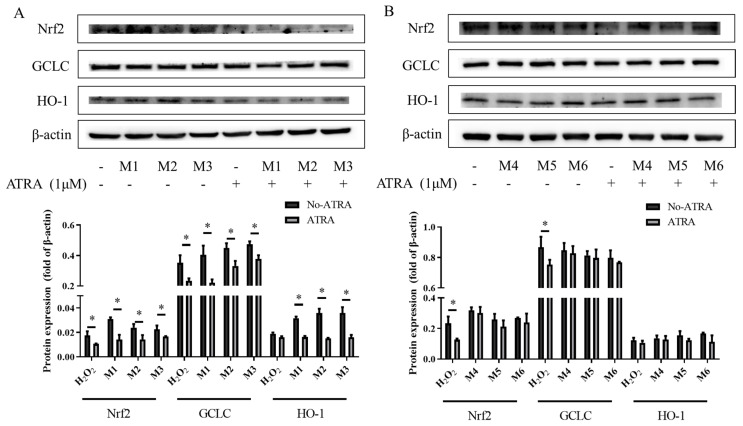
Effects of the pretreatments with all-trans retinoic acid (ATRA) and no ATRA in the M1, M2, M3, M4, M5, and M6 groups on Nrf2, guidance control launch console (GCLC), and Heme oxygenase 1 (HO-1) protein expression in H9c2 cells. (**A**) Cells treated with phytochemical combinations (M1, M2, and M3). (**B**) Cells treated with phytochemical combinations (M4, M5, and M6). All-trans retinoic acid (ATRA) was used to inhibit the Nrf2 pathway and observe the expression of Nrf2, HO-1, and GCLC. β-actin was used as the internal control for the Western blot assay. DP—delphinidin, CA—chlorogenic acid, LY—lycopene, LU—lutein, M1: CA-LU F3/10, M2: DP-CA F7/10, M3: DP-LY F5/10, M4: LY-LU F5/10, M5: CA-LU F9/10, M6: DP-LY F7/10. Where CA-LU F1/10 refers to the quantity of chlorogenic acid (1/10) to lutein (9/10) in the binary mixtures. * *p* < 0.05 indicated that the protein expression of the ATRA group was significantly different compared with the no-ATRA group.

ATRA significantly decreased the expression of Nrf2, HO-1, and GCLC in the M1, M2, and M3 groups (Figure 4, *p* < 0.05). This suggested that the expression of Nrf2 and its downstream antioxidant proteins in synergistic groups (M1, M2, and M3 groups) could be inhibited by ATRA. However, ATRA did not significantly change the protein expression of Nrf2 signaling in the M4, M5, or M6 groups (*p* < 0.05). The results indicated that there was no obvious activation of the Nrf2 pathway in the M4, M5, or M6 groups. The significant antagonistic effect on GCLC protein expression found in the M6 group may be due to the activation of other pathways (such as NF-κB). Based on the accumulation of Nrf2 in the nucleus, it could be concluded that the synergistic interactions of the M1, M2, and M3 groups were caused by the accumulation of Nrf2 in the nucleus, but the possible mechanism of the antagonistic interactions of the M4, M5, and M6 groups on protein expression was not through the activation of the Nrf2 pathway.

### 3.7. Interaction Effects of Phytochemical Combinations on Mitochondrial Membrane Potential

**Figure 5:**
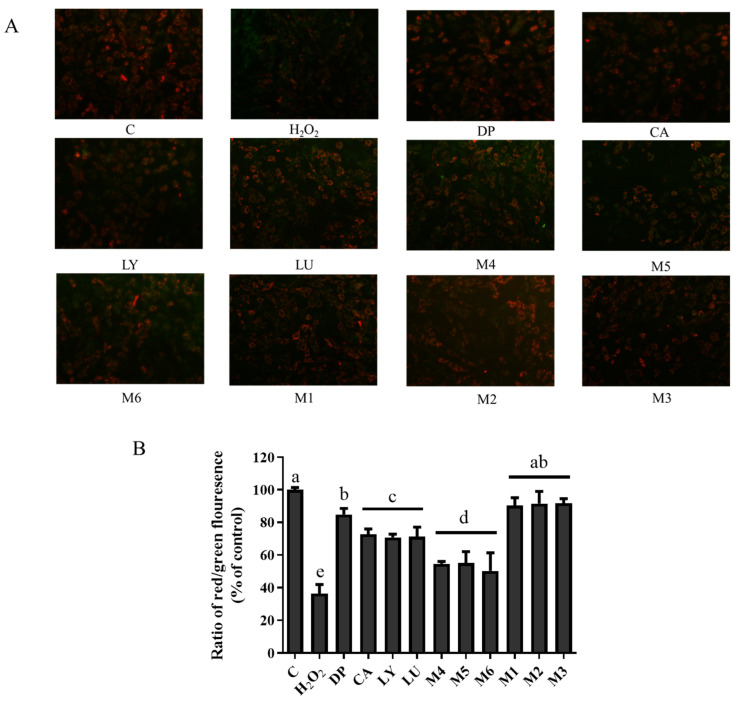
Effects of phytochemicals and their combinations on H_2_O_2_-induced dissipation of mitochondrial membrane potential (ΔΨm). (**A**) Morphology of H9c2 cells with or without pretreatment with phytochemicals. (**B**) The fluorescence intensity red-to-green ratio indicated a change in ΔΨm. JC-1 dye was added to determine the dissipation of ΔΨm. JC-1 identifies mitochondria exhibiting low membrane potentials by the emission of green fluorescence but also at high membrane potentials emitting a bright red-orange fluorescence. DP—delphinidin, CA—chlorogenic acid, LY—lycopene, LU—lutein, M1: CA-LU F3/10, M2: DP-CA F7/10, M3: DP-LY F5/10, M4: LY-LU F5/10, M5: CA-LU F9/10, M6: DP-LY F7/10. Where CA-LU F1/10 refers to the quantity of chlorogenic acid (1/10) to lutein (9/10) in the binary mixtures. Values with different letters (a–e) indicate that the membrane potentials of each group are significantly different (*p* < 0.05).

Many physiological and biochemical responses were dependent on the proper mitochondrial function, such as the production of ROS, the regulation of ATP level and calcium homeostasis, and the synthesis of amino acids. The low membrane potentials in JC-1 staining showed that the red fluorescence decreased, and the green fluorescence increased, which also indirectly reflected the damage process of the mitochondrial outer membrane. The red fluorescence decreased significantly in the H_2_O_2_-induced group compared with the control group (Figure 5). This indicated a decrease in mitochondrial membrane potential when the cells were induced by H_2_O_2_. However, the ratio of red to green fluorescence in the individual phytochemical groups was strikingly higher than that in the H_2_O_2_-induced group. It was noteworthy that the M1, M2, and M3 groups showed a higher ratio of red to green fluorescence, but the M4, M5, and M6 groups had lower values than the individual phytochemical groups (*p* < 0.05). 

### 3.8. The Effects of Phytochemical Combinations on Cell Absorption of Lycopene and Lutein

The initial concentration of LY and LU was 5 μM in the DMEM medium (Figure S6). The content of LY was 4.75 ± 0.00 μM after 2 h, showing no discernible change (*p* > 0.05). At 12 h, its content was 2.90 ± 0.00 μM, and the degradation was 41.95%. However, the content of LU was 4.67 ± 0.01 μM, and the degradation was 6.57% after 12 h. Thus, LU was more stable in the DMEM medium than LY.

In order to explore the interaction among the cell absorption of the four phytochemicals in the significantly synergistic and antagonistic groups, we detected the concentration of LY after cellular uptake per milligram of cellular protein, and three groups containing LY (M3: DP-LY = 1:1, M4: LY-LU = 1:1 and M6: DP-LY = 7:3) were selected for experiments. As shown in Table 2, except for M3, the absorption of LY in the LY group alone and the M4 and M6 groups with high concentration (5 μM) was higher than that in the low-concentration group (3 μM, *p* < 0.05), respectively. In the significant synergistic group (M3), the absorption of LY in the high and low-concentration groups were 0.92 ± 0.024 and 0.80 ± 0.075 (nmol/mg protein), which were significantly higher than that in the LY group (0.84 ± 0.018, 0.61 ± 0.007 nmol/mg protein, *p* < 0.05), respectively. Because of the synergism of DP, the cellular uptake of LY in the low-concentration group increased by 31.1%. However, in the significant antagonistic group (M4 and M6), the absorption of LY in the high and low-concentration groups was significantly lower than that in the LY group (*p* < 0.05). Furthermore, because of the antagonism of LU in M4, the absorption of LY in the high concentration was reduced by 43.3%. And because of the antagonism of DP in M6, the absorption of LY in the low concentration was reduced by 37.7%. Therefore, in the significant synergistic group, DP promoted the absorption of LY, while in the antagonistic group, LU and DP inhibited the absorption of LY, and the interaction of antagonism and synergism may be influenced by cell absorption.

Similarly, three significantly synergistic and antagonistic groups containing LU (M1: CA-LU = 3:7, M4: LY-LU = 1:1, and M5: CA-LU = 9:1) were also selected for experiments (Table 2). The absorption of LU in the LU and M4 groups with high concentrations (5 μM) was 0.18 ± 0.001 and 0.37 ± 0.007 (nmol/mg protein), respectively. It was higher than that in the low-concentration group (3 μM, 0.07 ± 0.002, 0.19 ± 0.006 nmol/mg protein, *p* < 0.05). In the high-concentration groups, the cellular uptake of LU in the M1 and M4 groups was 0.36 ± 0.024 and 0.37 ± 0.007 (nmol/mg protein), which was higher than that in the LU group (*p* < 0.05), while there was no significant difference in the M5 group. In the low-concentration groups, because of the effect of CA and LY, the absorption of LU in the M1, M4, and M5 groups was 1.85 times, 1.71 times, and 85% higher than that in the LU group (0.07 ± 0.002 nmol/mg protein, *p* < 0.05). Therefore, CA and LY promoted the cellular absorption of LU in both the synergistic and antagonistic groups

## 4. Discussion

### 4.1. Lycopene, Lutein, Chlorogenic Acid, and Delphinidin Showed Antioxidant Activities at Low Concentrations but Pro-Oxidant Activities at High Concentrations

LY, LU, CA, and DP are currently believed to exert cardioprotective effects in humans via their ability to reduce ROS. Based on this, and taking the human dietary pattern into consideration, the antioxidant interaction of their mixtures was studied in a cellular system. LY, LU, and CA showed antioxidant activity at low concentrations (LY < 3 μM, LU < 2.5 μM, CA < 18 μM), and the antioxidant activity decreased when the concentrations were further increased, which indicated that LY, LU, and CA had pro-oxidant effects at high concentrations. Notably, the antioxidant activity of DP was enhanced when its concentration increased from 0.5 µM to 10 µM. It has also been reported that the concentration of DP reached a certain level (>40 μM), which resulted in a decrease in the cellular antioxidant activity [14]. Similar results emerged that LY at 20 mM significantly enhanced the levels of TBARS induced by a lipid-soluble radical generator (2,2′-azobis [2,4-dimethylvaleronitrile]; AMVN). The antioxidant and pro-oxidant effects of LY tend to be dose-dependent [15]. Similarly, LU, CA, and DP show antioxidant and pro-oxidant behavior [16,17,18]. Epidemiological and experimental evidence suggest that phytochemicals are powerful antioxidants, but when phytochemicals lose an electron or act as reducing agents to become free radicals, their oxidation intermediates such as free radicals semiquinone and quinone in a high concentration lead to oxidation processes and severe damage to biological molecules, especially to DNA, lipids, and proteins [19]. The antioxidant and pro-oxidant bioactivities of phytochemicals also depend on the characteristics and utilities of free form. For example, lutein dimyristate (LD) tends to increase the oxidation of corn-triacylglyceride (TAG) at high concentrations, while having little effect at low concentrations. Nonetheless, free lutein (FL) tends to encourage the oxidation of corn-TAG, albeit at low concentrations because of the higher stability of FL16.

### 4.2. Antioxidant Interactions Were Influenced by the Effects of Combined Phytochemicals at Different Ratios on H9c2 Cells Damaged by Oxidation Stress

Primarily, it appears that some antioxidant interactions existed in the four phytochemicals evaluated by the generation of intracellular ROS and the quantification of CAA. For example, the F5/10 DP-LY, F7/10 DP-CA, and F5/10 CA-LY groups, and the F3/10 CA-LU group in particular, showed synergistic interaction with the CI values of 0.57 ± 0.04, 0.53 ± 0.01, 0.94 ± 0.25 and 0.27 ± 0.15, respectively. In addition, the F7/10 DP-LY, F9/10 CA-LU, and F1/10 DP-LU groups, especially the F5/10 LY-LU group, showed antagonistic interaction with CI values of 4.54 ± 1.52, 6.40 ± 5.01, 4.30 ± 1.29, and 22.85 ± 1.93, respectively. According to the CI_25_ value, M1 (F3/10 CA-LU), M2 (F7/10 DP-CA), and M3 (F5/10 DP-LY) groups were selected as the significant antioxidant synergistic groups. Likewise, the M4 (F5/10 LY-LU), M5 (F9/10 CA-LU), and M6 (F7/10 DP-LY) groups were determined as antagonistic groups. It is remarkable that antioxidant interactions of phytochemicals were different because of the different ratios of combinations. 

Generally, when phytochemicals are mixed, their disparate antioxidant activities for scavenging ROS in cells may be influenced by their distinct structures. CA contains an o-diphenol hydroxyl group, and DP contains three ortho hydroxyl groups on the B ring. The hydroxyl structural properties seem to affect their antioxidant activities [20]. Three primary mechanisms—electron transfer, allylic hydrogen abstraction, and radical addition to the conjugated double bond system—allow carotenoids, chain-breaking antioxidants, to scavenge ROO• [21]. The chromophore extension (number of conjugated double bonds) and the opening of the β-ionone ring of carotenoids appear to have the greatest effects on the ability to scavenge ROO• (a kind of ROS produced by the breakdown of hydroperoxides) [22]. However, another study found that LY and LU did not show an obvious antioxidant effect in experiments when ROO• was generated by AAPH [23]. As reported in another paper, all of the combinations showed antagonism at a 1:1 ratio of six common anthocyanidins to LY but showed an additive effect at a 3:1 ratio of LY to anthocyanins in Caco-2 cells [24]. Conversely, all groups of lutein–anthocyanin combinations at 1:1, 1:3, and 3:1 ratios showed additive CAA in Caco-2 cells [25]. Our previous study [26] found that hydrophilic phenolic and lipophilic carotenoids combined at different ratios exhibited different interactions. Therefore, it followed that the interaction between carotenoids and phenolics did not seem to correlate significantly with their ratios. In other words, it was speculated that given a balanced ratio, the health benefits of a certain ratio of ingredients among daily foods may be better than some individual foods.

### 4.3. The Expression of Antioxidant Enzymes May Not Be Consistent with the Changes in Antioxidant Activities Caused by the Interactions of Phytochemical Combinations

In the present work, the generation of intracellular ROS and the expression of antioxidant enzymes were used to further study the antioxidant interactions of phytochemical combinations. In addition, CAA was used to detect the level of ROS in cells within 1 h. The settings of the CAA tests were similar to biological systems, making them more suitable methodologies for assessing the antioxidant activity of phytochemicals [11]. In the CAA assays, the CI_25_ values of the M1, M2, and M3 groups were less than 1, which meant that they had strong synergistic effects on reducing the level of ROS compared with the individual groups. In addition, the M4, M5, and M6 groups showed good antagonistic effects. The CAA results were in agreement with the results of the flow cytometric detection of intracellular ROS generation in H9c2 cells.

Intracellular antioxidant defense consists of several key enzymes (SOD, GSH-PX, and CAT). Phytochemicals could enhance the expression of antioxidant enzymes to eliminate ROS [27]. Hence, in order to investigate the antioxidant interactions of phytochemical combinations, the activities of several antioxidant enzymes (GSH-Px, SOD, and CAT) in H_2_O_2_-induced H9c2 cells were evaluated. However, it was noticed that the interactional effects on the expression of enzymes (SOD, GSH-Px, and CAT) in the six groups were inconsistent with other indicators (the inhibition of ROS production and the quantification of CAA). For example, the expression of SOD activity in the M2 group showed a significant synergistic effect (*p* < 0.05), while the expressions of GSH-Px and CAT in the M2 group were basically in line with the expected activity and showed an additive effect. What is more, a synergistic interaction was detected in the M4 group in the activity of CAT (*p* < 0.05). However, the interactive effect on the activity of GSH-Px in the M4 group was antagonistic. This is possibly because of the following: (1) carotenoids and antioxidant enzymes existed the specific interaction. Carotenoids probably lowered the activity of SOD, which is the first step in the detoxification chain of superoxide radicals, while promoting the activity of CAT, which is the second step in the detoxification chain [28]. The interaction may have caused the M1, M2, M3, M4, M5, and M6 groups to show a more significant effect on the activities of antioxidant enzymes (SOD, GSH-Px, CAT). (2) Even if the antioxidant activities of some phytochemicals were related to the antioxidant defense system including antioxidant enzymes (CAT, SOD, and GSH-Px), the reaction substrate, catalytic properties, and physical and chemical properties were different, and the synergistic and antagonistic activities of binary combinations were diverse in different enzyme systems. This made it difficult for phytochemical combinations to perform as expected. As reported in another paper, in the inhibition experiment of α-amylase, the best combination was EGCG: GSP = 8:2, and the synergistic rate was 36.08%. However, the combination had no significant synergistic activity in the inhibition of α-glucosidase. It was clear that the antioxidant enzymes may be inconsistent with the changes in antioxidant activities caused by interactions of phytochemical combinations.

### 4.4. TheAntioxidant Interaction of Synergistic Groups (M1, M2, and M3 Groups) Were Mediated by the Activation of the Nrf2 Pathway in H9C2 Cells

**Figure 6:**
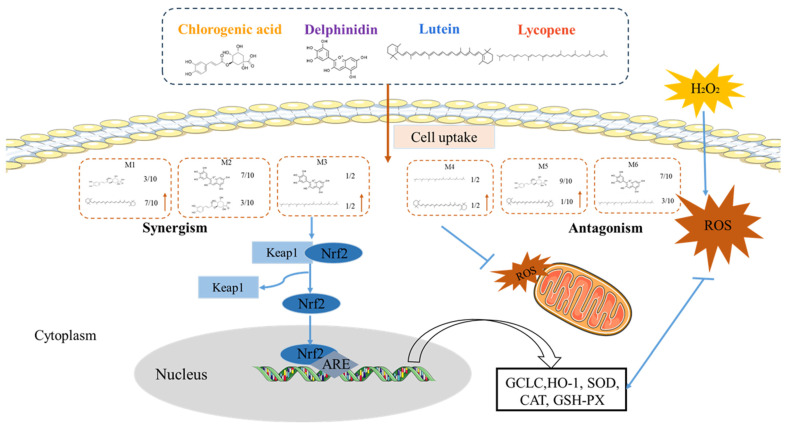
Proposed mechanisms of antioxidant interactions among phytochemical combinations.

The Keap1-Nrf2 pathway is the primary regulator of cytoprotective responses to oxidative stress (Figure 6). It was reported that numerous antioxidant proteins, such as HO-1 and GCLC, are controlled by Nrf2 signaling pathways. Oxidative stress will release Nrf2 from Keap1 and permit the translocation of Nrf2 to the nucleus, where Nrf2 interacts with the antioxidation response element ARE that triggers the expression of antioxidant proteins including HO-1 and GCLC [29,30]. 

The Western blot analysis revealed that in the M1, M2, and M3 groups, there was a significant synergistic rise in the protein levels of GCLC and HO-1. Consequently, for the accumulation of Nrf2 in the nucleus, our data showed that the pretreatment of phytochemical mixtures in the M1, M2, and M3 groups significantly restored the nuclear translocation of Nrf2 in the H_2_O_2_-induced H9c2 cells. The M4, M5, and M6 groups downregulated GCLC and HO-1 expression; however, they did not enhance the accumulation of Nrf2 in the nucleus. The experiment aimed to explore the inhibitive function of ATRA on the Nrf2 pathway in the six study groups, and it showed that ATRA significantly decreased the co-expression of Nrf2, HO-1, and GCLC in the M1, M2, and M3 groups compared with the no-ATRA groups. It is worth noting that ATRA did not trigger the expression changes in Nrf2, HO-1, or GCLC in the M4, M5, and M6 groups compared with the no-ATRA groups. Previous studies found that LY [31], LU [32], CA [33], and DP [34] can protect cells from oxidant damage separately through the action of the Nrf2 pathway. The possible mechanism underlying the synergistic antioxidant interactions of phytochemical combinations in the M1, M2, and M3 groups may be that phytochemical combinations activate the Nrf2 pathway to protect H9c2 cells damaged by oxidative stress. In contrast, the protein expression in the M4, M5, and M6 groups was not mediated by the activation of the Nrf2 pathway. 

The antioxidant mechanisms underlying the cellular bioactivities of a single phytochemical can be studied. However, a detailed understanding of the molecular mechanisms underlying the bioactivity interactions among phytochemicals is difficult to achieve. One constraining issue is the discrepancy between the predicted expression of a molecular pathway, which stems from the cumulative impact of its constituent phytochemical components, and the actual expression, which is determined by the interaction of different phytochemical combinations.

### 4.5. Mitochondria Are a Primary Target of Antioxidant Interactions of Phytochemical Mixtures

Mitochondria play a central role in energy generation and complex processes such as apoptosis and cardio protection within the cell [35]. Mitochondria, being the main site of ROS generation in the cell, are also their primary target. Mitochondrial DNA mutations may initiate peroxidation, proteins, lipids, and opening of the mitochondrial permeability transition pore, which is linked to an increase further in ROS generation and a decrease in membrane potential across mitochondria. This, in turn, leads to apoptotic cell death [36,37,38]. In our study, the free radicals produced by H_2_O_2_ led to a decrease in membrane potentials in cells [35]. However, the cells treated with phytochemicals could inhibit this decrease. Phytochemicals decrease the accumulation of ROS in mitochondria. This, in turn, results in less damage to the mitochondrial respiratory chain and relatively high membrane potentials. In this study, phytochemical combinations (M1, M2, and M3 groups) exhibited relatively higher membrane potentials compared with the individual groups. Conversely, the M4, M5, and M6 groups had lower membrane potentials compared with the individual groups. The interactions (synergistic or antagonistic) of phytochemical combinations in membrane potentials across mitochondria corresponded with the generation of ROS detected by CAA and flow cytometry. Thus, the antioxidant interaction (synergistic or antagonistic) of phytochemical combinations may be located in mitochondria. That is to say, mitochondria were the main site of ROS generation in the cell, which were also the primary target of antioxidant interactions of the phytochemical mixtures.

This paper showed that some combinations (M1: CA-LU: F3/10, M2: DP-CA: F7/10, M3: DP-LY: F5/10) had stronger synergistic antioxidant effects than the individual groups. And others showed antagonistic effects (M4: LY-LU: F5/10, M5: CA-LU: F9/10, M6: DP-LY: F7/10). However, the expression of antioxidant enzymes may not be consistent with the changes in antioxidant activities caused by the interactions of phytochemical combinations. The possible mechanism underlying the synergistic interactions of phytochemical combinations on antioxidant activities may be developed by upregulating the expression of the Nrf2 pathway. Mitochondria play a central role in the antioxidant interaction of the phytochemical mixtures.

### 4.6. The Stability of Carotenoids in the Medium Doe Not Directly Affect Cell Absorption, and the Absorption of Phytochemicals Is Closely Related to Antioxidant Interactions

The stability of LY and LU in the DMEM culture was determined by HPLC, and the effect of another component in the phytochemical combination on LY and LU was investigated. The results showed that LU was more stable in the DMEM medium, while LY was less stable. Nevertheless, the absorption of LY was higher than that of LU when the initial concentration of both was 5 μM or 3 μM. This suggests that the cellular absorption of carotenoids may be closely related to the carotenoid transporters. With the increase in culture time, the carotenoid content in the medium was lost to some extent, and some of it was absorbed by cells. Finally, the concentration of carotenoid measured by HPLC was only a few percent of the initial concentration. Hence, the stability of carotenoids in the medium does not directly affect cell absorption.

In the synergistic and antagonistic groups (M1 and M4 groups), CA and LY both promoted the absorption of LU. In the synergistic group (M3 group), DP promoted the absorption of LY, while in the antagonistic group (M4 and M6 groups), LU and DP inhibited the absorption of LY. Therefore, the significant synergistic and antagonistic effects may be influenced by interactions in cellular absorption. According to Kirakosyan, in some antioxidant groups, total biological activity may be inhibited by reducing the stability or bioavailability of one component or by enhancing its metabolism [39]. In this experiment, the absorption of LU was promoted in both the synergistic and antagonistic groups. It can be inferred that the absorption of LU in the synergistic group (M1) may be improved by promoting the bioavailability of LU. In the significant antagonistic group (M1 and M4), LY and CA also promoted the absorption of LU. On the other hand, with the accumulation of LU and its oxidation products, the intracellular antioxidant pathway was altered, and the expression of antioxidant proteins was reduced, which caused the pro-oxidative effect. When the pro-oxidation of cumulative effect was more powerful than the antioxidation of the combined groups, it showed antioxidant antagonism. Above all, the absorption of phytochemicals was closely related to antioxidant interactions.

## 5. Conclusions

In this paper, it was shown that some combinations of lycopene, lutein, chlorogenic acid, and delphinidin (M1: CA-LU: F3/10, M2: DP-CA: F7/10, M3: DP-LY: F5/10) exhibited stronger synergistic antioxidant effects than the individual groups. And others showed antagonistic effects (M4: LY-LU: F5/10, M5: CA-LU: F9/10, M6: DP-LY: F7/10). However, the expression of antioxidant enzymes may not be consistent with the changes in antioxidant activities caused by interactions of phytochemical combinations. The possible mechanism underlying the synergistic interactions of the phytochemical combinations on antioxidant activities may be developed by upregulating the expression of the Nrf2 pathway. Mitochondria play a central role in the antioxidant interaction of the phytochemical mixtures. In addition, the enhancement in or suppression of intracellular compound contents by other compounds may result in synergy or antagonism on the cellular antioxidant activities of the combinations. Our study contributes not only to providing a theoretical basis for the research of functional foods and reasonable diet collocation but also to encouraging the continued advancement of phytochemicals in the fields of food chemistry and pharmacy.

## Figures and Tables

**Table 1:** Combination index and interactive effects of binary combinations on cellular antioxidant activity ^1^.

Combination of Materials ^2^	Fraction ^3^	CI_25_^4^
DP-LY	F 1/10	0.91 ± 0.08 ^b^
	F 3/10	0.91 ± 0.13 ^b^
	F 5/10	0.57 ± 0.04 ^b^
	F 7/10	4.54 ± 1.52 ^a^
	F 9/10	0.75 ± 0.18 ^b^
CA-LU	F 1/10	0.88 ± 0.21 ^ab^
	F 3/10	0.27 ± 0.15 ^a^
	F 5/10	0.85 ± 0.16 ^ab^
	F 7/10	0.71 ± 0.01 ^ab^
	F 9/10	6.40 ± 5.01 ^a^
CA-LY	F 1/10	0.84 ± 0.09 ^ab^
	F 3/10	0.74 ± 0.18 ^b^
	F 5/10	0.94 ± 0.25 ^ab^
	F 7/10	2.38 ± 1.27 ^a^
	F 9/10	1.07 ± 0.05 ^ab^
DP-CA	F 1/10	1.16 ± 0.16 ^a^
	F 3/10	0.62 ± 0.12 ^b^
	F 5/10	0.96 ± 0.34 ^ab^
	F 7/10	0.53 ± 0.01 ^b^
	F 9/10	0.67 ± 0.02 ^b^
DP-LU	F 1/10	4.3 ± 1.29 ^a^
	F 3/10	0.97 ± 0.36 ^b^
	F 5/10	0.71 ± 0.04 ^b^
	F 7/10	2.19 ± 0.72 ^b^
	F 9/10	1.35 ± 0.14 ^b^
LY-LU	F 1/10	2.46 ± 1.93 ^c^
	F 3/10	14.33 ± 0.75 ^b^
	F 5/10	22.85 ± 1.93 ^a^
	F 7/10	1.35 ± 0.67 ^c^
	F 9/10	0.59 ± 0.19 ^c^

^1^ Values with different letters (a–c) in the same column indicate that the CI_25_ values of the phytochemical combinations at different ratios are significantly different (*p* < 0.05). ^2^ DP-LY, delphinidin and lycopene; CA-LU, chlorogenic acid and lutein; CA-LY, chlorogenic acid and lycopene; DP-CA, delphinidin and chlorogenic acid; DP-LU, delphinidin and lutein; LY-LU, lycopene and lutein. ^3^ F1/10 (DP-LY) refers to the quantity of delphinidin (1/10) to lycopene (9/10) in the binary mixtures. ^4^ CI_25_ means the CI value at the scavenging capacity of 25%. The CI value was calculated automatically by CompuSyn (version 1.0).

**Table 2:** Absorption ^1^ of lycopene and lutein by HPLC.

Group	Compound ^2^	Low Concentration (nmol/mg Protein) ^3^	High Concentration (nmol/mg Protein) ^4^
LY	LY	0.61 ± 0.007 ^b^	0.84 ± 0.018 ^b,^*
	M3	0.80 ± 0.075 ^a^	0.92 ± 0.024 ^a^
	M4	0.38 ± 0.001 ^c^	0.62 ± 0.04 ^c,^*
	M6	0.40 ± 0.006 ^c^	0.57 ± 0.011 ^c,^*
LU	LU	0.07 ± 0.002 ^b^	0.18 ± 0.001 ^b,^*
	M1	0.20 ± 0.004 ^a^	0.36 ± 0.024 ^a^
	M4	0.19 ± 0.006 ^a^	0.37 ± 0.007 ^a,^*
	M5	0.13 ± 0.006 ^a^	0.19 ± 0.023 ^b^

Values are mean ± SD (*n* = 3). * A significant difference (*p* < 0.05) was seen between the high-concentration group and the low-concentration group. ^a,b,c^ Different letters indicate a significant difference (*p* < 0.05) in cellular uptake of lycopene or lutein seen in different combinations at the same concentrations. ^1^ Absorption means the concentration of lycopene or lutein after cellular uptake per milligram of cellular protein. ^2^ LY: lycopene only, LU: lutein only, M1: CA-LU = 3:7, M3: DP-LY = 1:1, M4: LY-LU = 1:1, M5: CA-LU = 9:1, M6: DP-LY = 7:3. ^3,4^ Lycopene and lutein in the low-concentration group was 3 μM, and lycopene and lutein in the high-concentration group was 5 μM.

## Data Availability

The original contributions presented in the study are included in the article/Supplementary Materials, further inquiries can be directed to the corresponding author.

## Supplementary Materials

The following supporting information can be downloaded at: https://www.mdpi.com/article/10.3390/foods13152440/s1, Figure S1: Effect of lycopene (LY), lutein (LU), chlorogenic acid (CA) and delphinidin (DP) on H9c2 cell viability. Significant difference compared with control group were indicated as * *p* < 0.05, ** *p* < 0.01. Figure S2: Generation of intracellular ROS in H9c2 cells treated with individual lycopene (LY), lutein (LU), chlorogenic acid (CA) and delphinidin (DP). Significant difference compared with H_2_O_2_ group were indicated as * *p* < 0.05; ** *p* < 0.01, *** *p* < 0.001, Figure S3: H_2_O_2_-induced oxidation of DCFH to DCF in H9c2 cells and the inhibition of oxidation by (A) lycopene (LY), (B) lutein (LU), (C) chlorogenic acid (CA) and (D) delphinidin (DP). CAA units can be calculated by this equation: CAA unit = 1 − (∫ SA/∫ CA), Figure S4: Generation of intracellular ROS in H9c2 cells treated with phytochemicals and binary combinations. DP—delphinidin, CA—chlorogenic acid, LY—lycopene, LU—lutein, M1: CA-LU F3/10, M2: DP-CA F7/10, M3: DP-LY F5/10, M4: LY-LU F5/10, M5: CA-LU F9/10, M6: DP-LY F7/10. Such as, CA-LU F1/10 refers to the quantity of chlorogenic acid (1/10) to lutein (9/10) in the binary mixtures. Values with different letters (a-c) showed the fluorescence intensity of each group was significant different (*p* < 0.05). Columns marked with an asterisk indicated that the experimental activities of the mixtures (M1, M2, M3, M4, M5, and M6 groups) were significantly different from the calculated additive values (*p* < 0.05). The horizontal lines showed the expected additive activities of the mixtures, which were calculated as RA/(P1 + RA/RB × P2) in which RA represented the ROS level of a single phytochemical A, RB represented the ROS level of a single phytochemical B, P1 represented the proportion of A, and P2 represented the proportion of B in the combinations. * indicated that the experimental value was significantly different from the theoretical value (*p* < 0.05), Figure S5: Effects of phytochemicals and binary combinations on SOD (A), GSH-PX (B) and CAT (C) activities induced by H_2_O_2_ in H9c2 cells. DP—delphinidin, CA—chlorogenic acid, LY—lycopene, LU—lutein, M1: CA-LU F3/10, M2: DP-CA F7/10, M3: DP-LY F5/10, M4: LY-LU F5/10, M5: CA-LU F9/10, M6: DP-LY F7/10. Such as, CA-LU F1/10 referred to the quantity of chlorogenic acid (1/10) to lutein (9/10) in the binary mixtures. Values with different letters (a-c) showed the activities of antioxidant enzymes of each group were significant different (*p* < 0.05). Columns marked with an asterisk indicated that the experimental activities of the mixtures (M1, M2, M3, M4, M5, and M6 groups) were significantly different from the calculated additive values (*p* < 0.05). The horizontal lines showed the expected additive activities of the mixtures, which were calculated as RA/(P1 + RA/RB × P2) in which RA represented the enzyme activity of a single phytochemical A, RB represented the enzyme activity of a single phytochemical B, P1 represented the proportion of A, and P2 represented the proportion of B in the combinations. * indicated that the experimental value was significantly different from the theoretical value (*p* < 0.05) Figure S6: The liquid phase signal strength and content of lycopene (A, B) and lutein (C, D) at different treatment time.
