# Supplementary material for: Synergistic and Antagonistic Activity of Selected Dietary Phytochemicals against Oxidative Stress-Induced Injury in Cardiac H9c2 Cells via the Nrf2 Signaling Pathway
Source: Foods. 2024 Aug 2;13(15):2440. doi: 10.3390/foods13152440 (PMC11312280; doi:10.3390/foods13152440)
Supplement: Supplementary file 1 [file foods-13-02440-s001.zip › foods-3104056-supplementary.pdf]

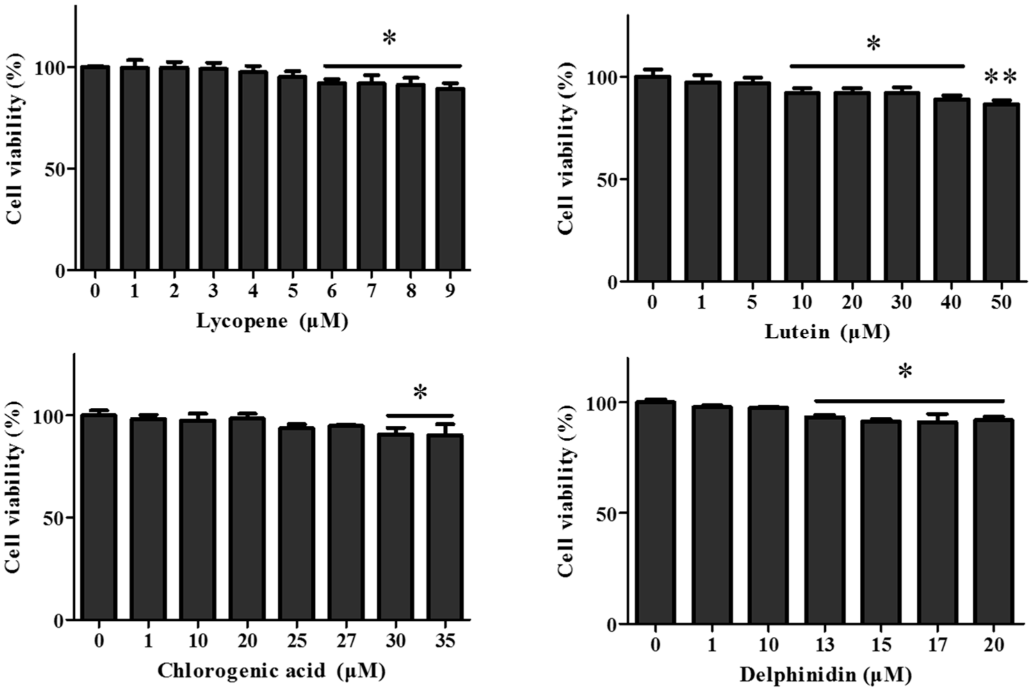

Figure S1 Effect of lycopene (LY), lutein (LU), chlorogenic acid (CA) and delphinidin (DP) on H9c2 cell viability. Significant difference compared with control group were indicated as* $p < 0.05$, ** $p < 0.01$.

A

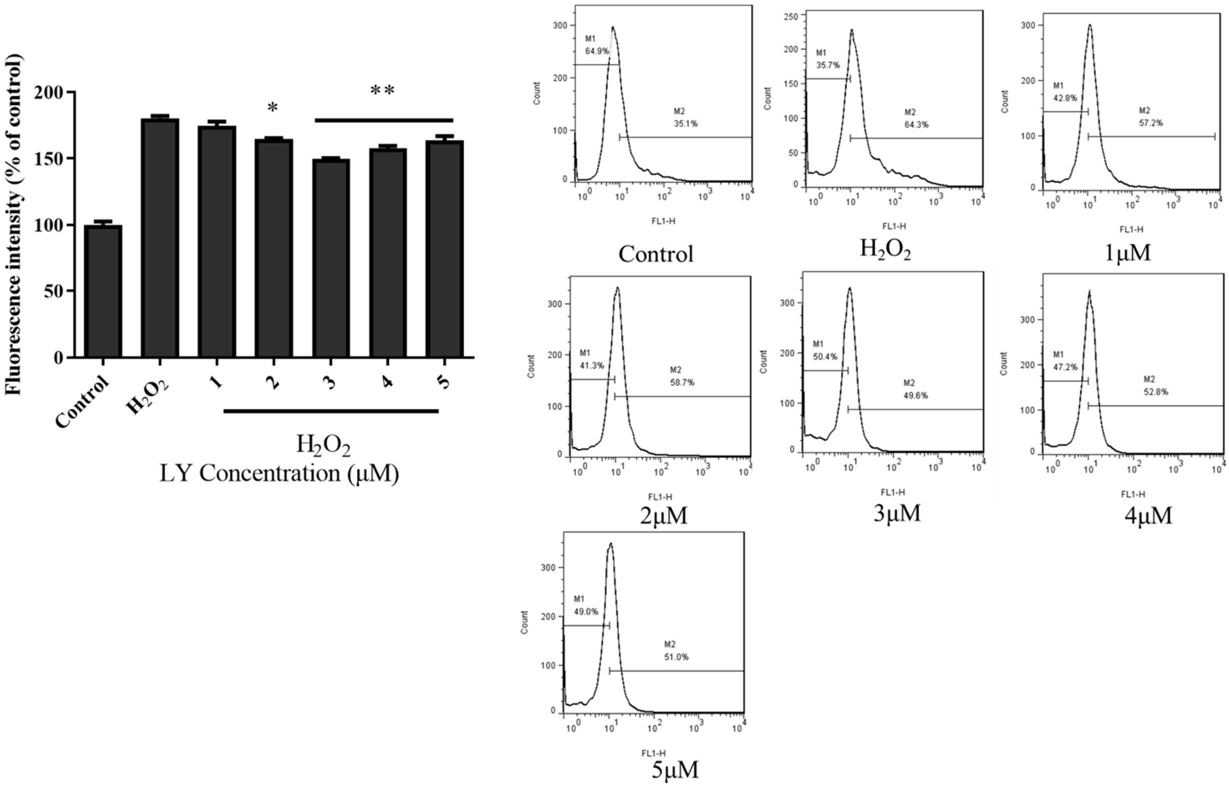

B

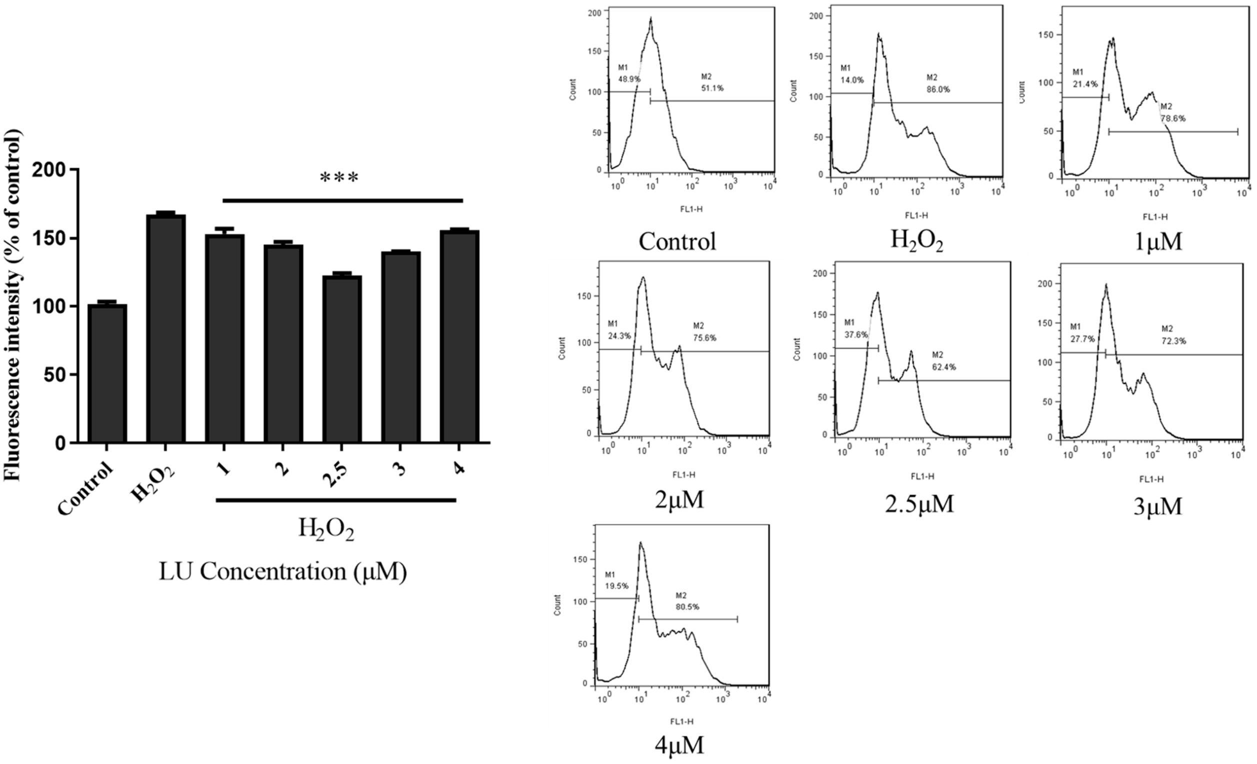
Fluorescence intensity (% of control)
***
Control
H2O2
1
2
2.5
3
4
H2O2
LU Concentration (μM)
Control
H2O2
1μM
2μM
2.5μM
3μM
4μM

C

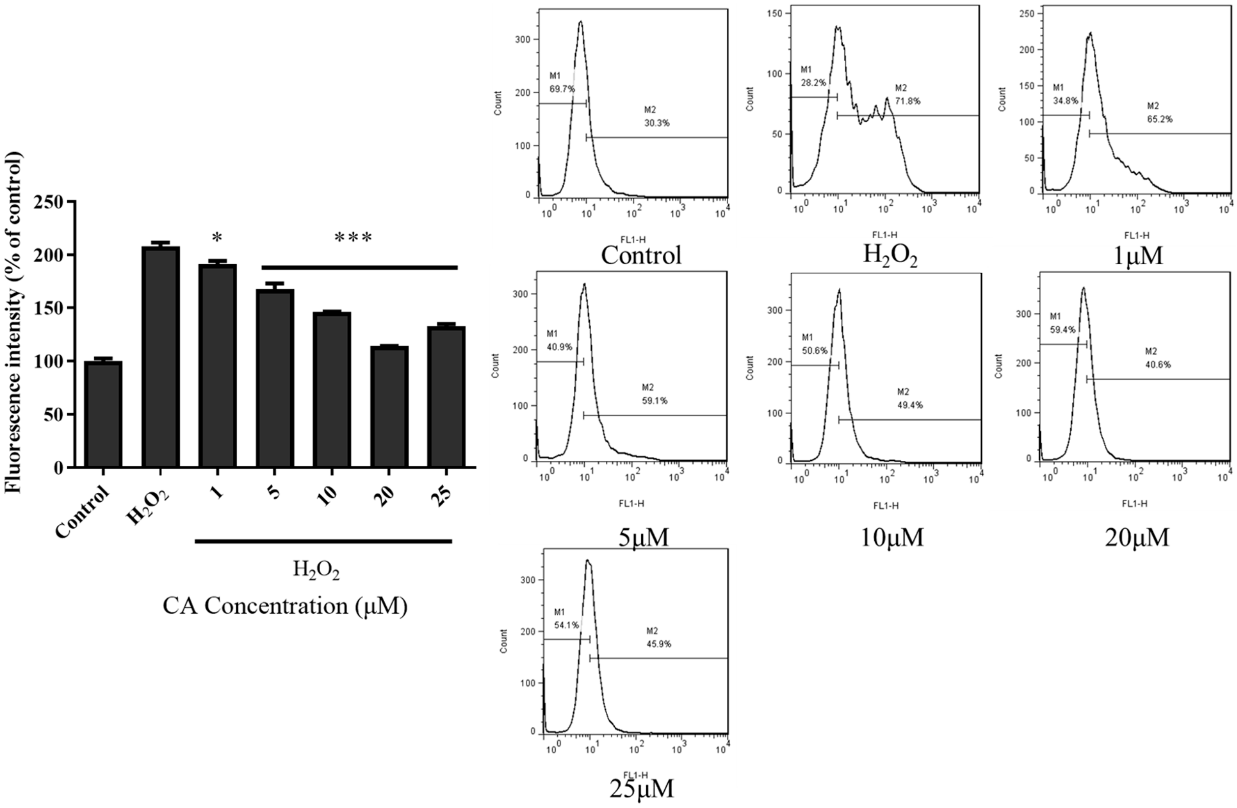
Fluorescence intensity (% of control)
*
***
Control
H2O2
1
5
10
20
25
H2O2
CA Concentration (μM)
Control
H2O2
1μM
5μM
10μM
20μM
25μM

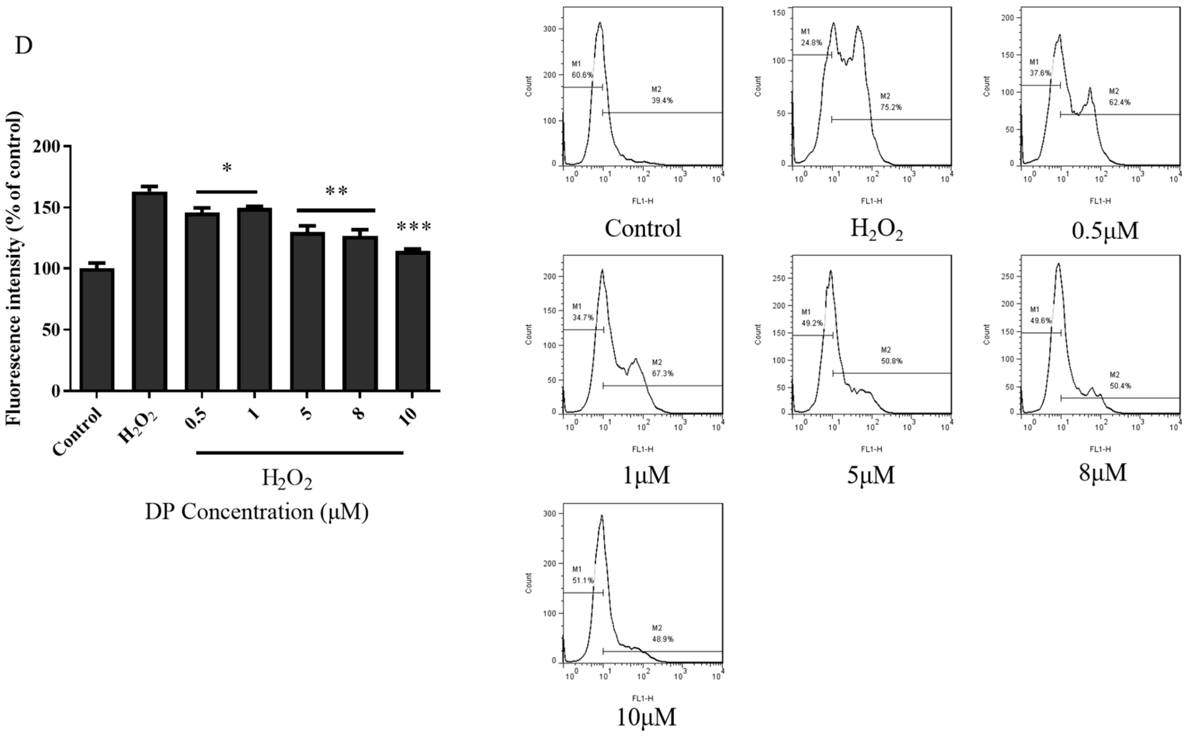

Figure S2 Generation of intracellular ROS in H9c2 cells treated with individual(A) lycopene (LY), (B) lutein (LU), (C) chlorogenic acid (CA) and (D) delphinidin (DP). Significant difference compared with H_2O_2 group were indicated as* $p < 0.05$; ** $p < 0.01$, *** $p < 0.001$.

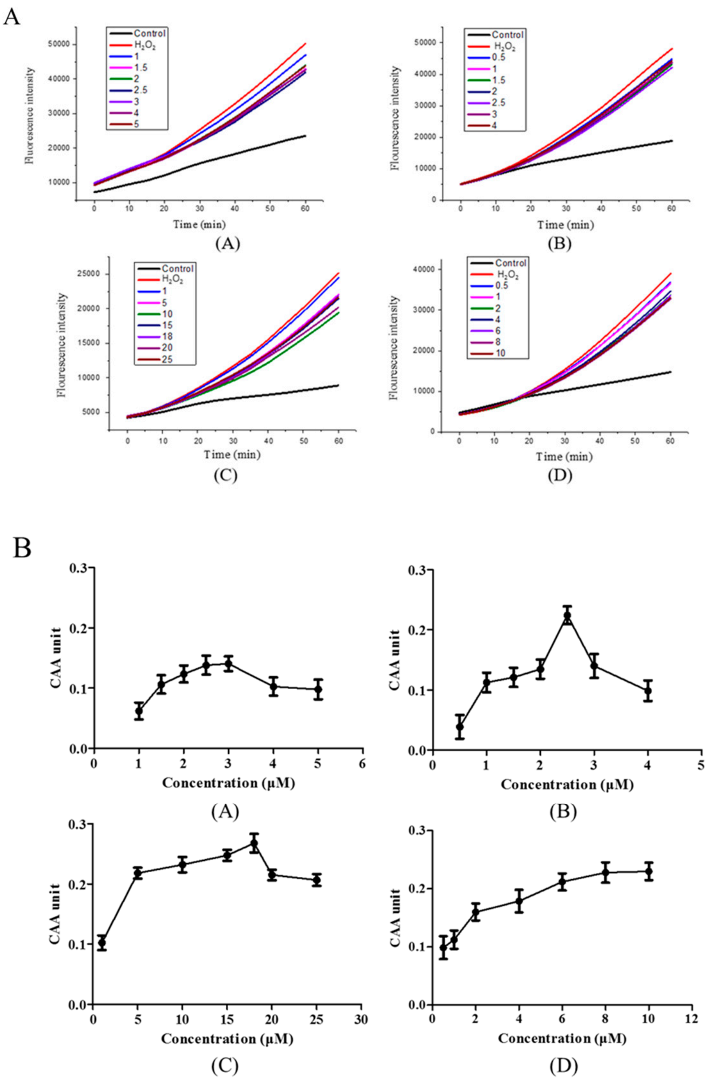

Figure S3 H_2O_2-induced oxidation of DCFH to DCF in H9c2 cells and the inhibition of oxidation by (A) lycopene (LY), (B) lutein (LU), (C) chlorogenic acid (CA) and (D) delphinidin (DP). CAA units can be calculated by this equation: CAA unit = 1 - (∫SA/∫CA).

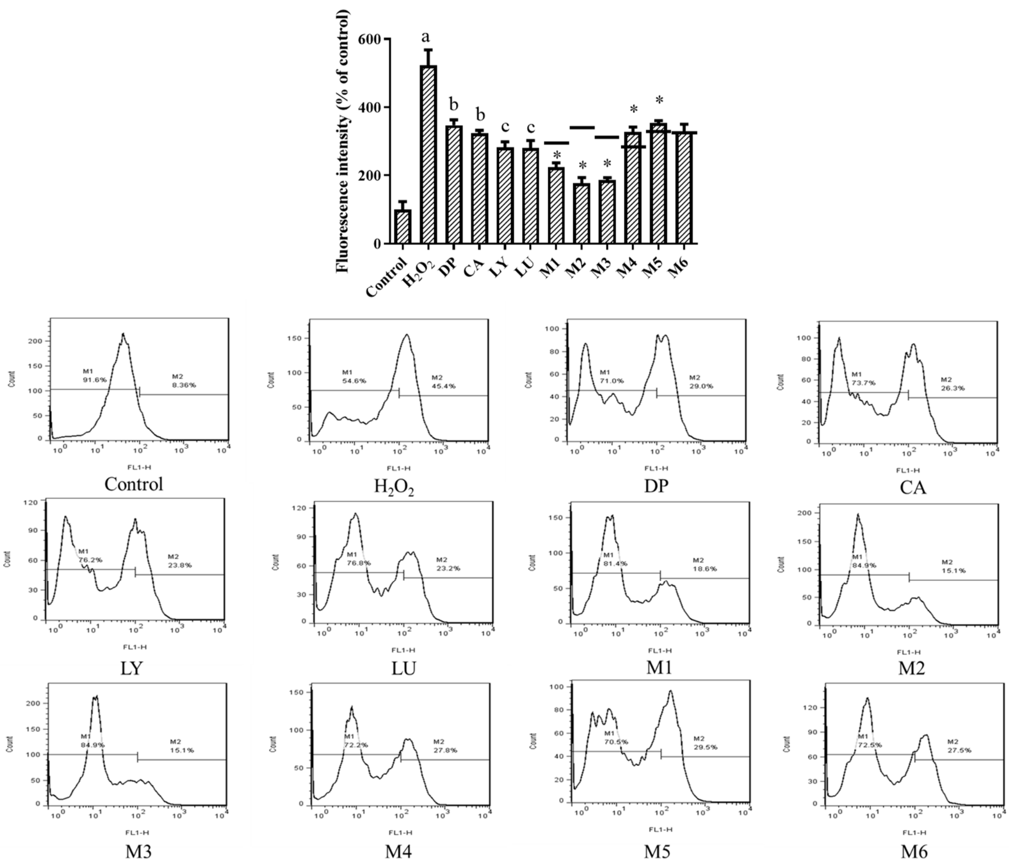

Figure S4 Generation of intracellular ROS in H9c2 cells treated with phytochemicals and binary combinations. DP-delphinidin, CA-chlorogenic acid, LY-lycopene, LU-lutein, M1: CA-LU F3/10, M2: DP-CA F7/10, M3: DP-LY F5/10, M4: LY-LU F5/10, M5: CA-LU F9/10, M6: DP-LY F7/10. Such as, CA-LU F1/10 refers to the quantity of chlorogenic acid (1/10) to lutein (9/10) in the binary mixtures. Values with different letters (a-c) showed the fluorescence intensity of each group was significant different ($p < 0.05$). Columns marked with an asterisk indicated that the experimental activities of the mixtures (M1, M2, M3, M4, M5, and M6 groups) were significantly different from the calculated additive values ($p < 0.05$). The horizontal lines showed the expected additive activities of the mixtures, which were calculated as RA/ (P1+RA/RB × P2) in which RA represented the ROS level of a single phytochemical A, RB represented the ROS level of a single phytochemical B, P1 represented the proportion of A, and P2 represented the proportion of B in the combinations. * indicated that the experimental value was significantly different from the theoretical value ($p < 0.05$).

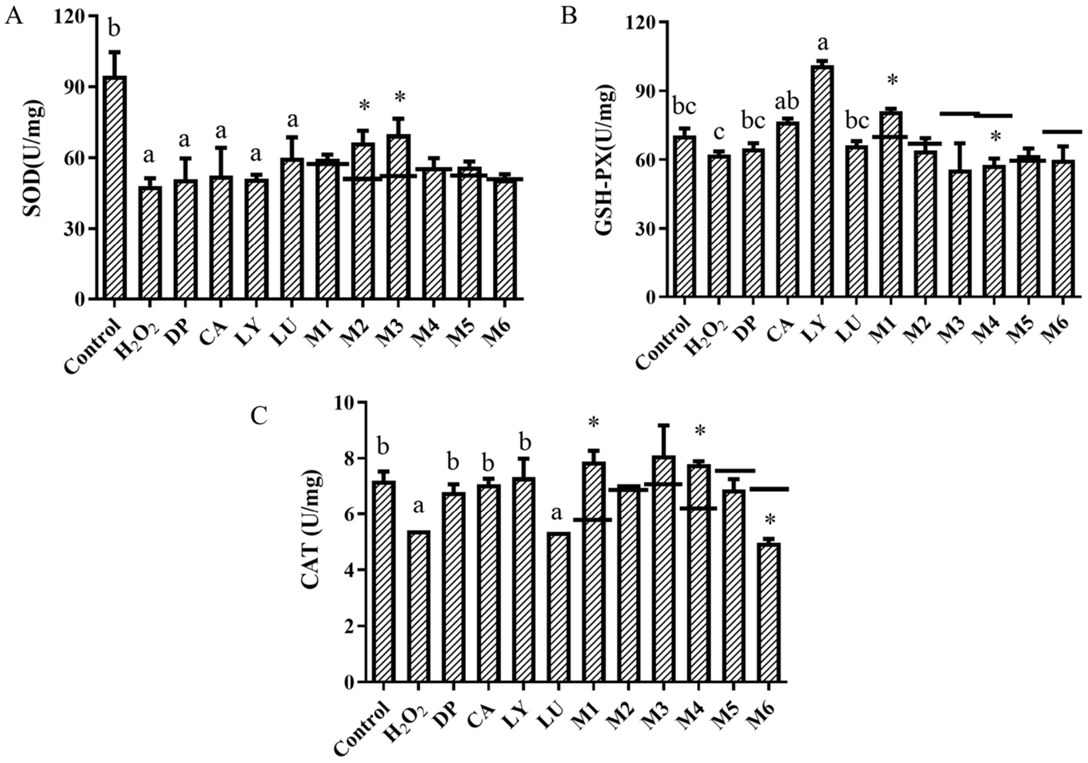

Figure S5 Effects of phytochemicals and binary combinations on SOD (A), GSH-PX (B) and CAT (C) activities induced by H_2O_2 in H9c2 cells. DP-delphinidin, CA-chlorogenic acid, LY-lycopene, LU-lutein, M1: CA-LU F3/10, M2: DP-CA F7/10, M3: DP-LY F5/10, M4: LY-LU F5/10, M5: CA-LU F9/10, M6: DP-LY F7/10. Such as, CA-LU F1/10 referred to the quantity of chlorogenic acid (1/10) to lutein (9/10) in the binary mixtures. Values with different letters (a-c) showed the activities of antioxidant enzymes of each group were significant different ($p < 0.05$). Columns marked with an asterisk indicated that the experimental activities of the mixtures (M1, M2, M3, M4, M5, and M6 groups) were significantly different from the calculated additive values ($p < 0.05$). The horizontal lines showed the expected additive activities of the mixtures, which were calculated as $RA/(P1+RA/RB \times P2)$ in which RA represented the enzyme activity of a single phytochemical A, RB represented the enzyme activity of a single phytochemical B, P1 represented the proportion of A, and P2 represented the proportion of B in the combinations. * indicated that the experimental value is significantly different from the theoretical value ($p < 0.05$).

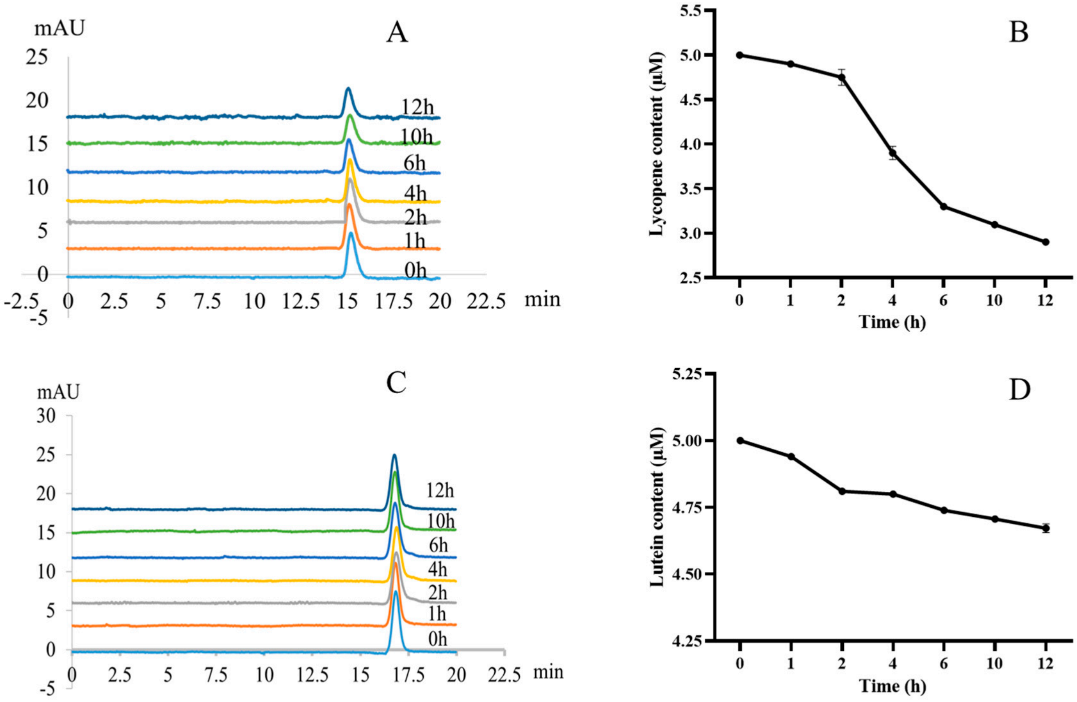

Figure S6 The liquid phase signal strength and content of lycopene (A, B) and lutein (C, D) at different treatment time.
